# Independent and dynamic manipulation of surface waves radiation for quadruplex polarization channels enabled by programmable coding metasurface

**DOI:** 10.1515/nanoph-2023-0820

**Published:** 2024-02-23

**Authors:** Zhenxu Wang, Tonghao Liu, Jian-Gang Liang, Jiafu Wang, Yueyu Meng, Xinmin Fu, Hongya Chen, Ruichao Zhu, Zuntian Chu, Yina Cui, Huiting Sun, Shaojie Wang, Hua Ma, Shaobo Qu

**Affiliations:** Shaanxi Key Laboratory of Artificially-Structured Functional Materials and Devices, Air Force Engineering University, Xi’an, Shaanxi 710051, China; Air and Missile Defense College, Air Force Engineering University, Xi’an, Shaanxi 710051, People’s Republic of China; Zhijian Laboratory, Rocket Force University of Engineering, Xi’an 710025, China

**Keywords:** surface waves radiation, quadruplex channels, programmable coding metasurface, independent and dynamic manipulation, high-integration intelligent metadevices

## Abstract

Flexible manipulation of surface waves (SWs) radiation has been continuously intriguing enormous interests of researchers due to its promising application prospects, and metasurfaces exhibit unparalleled capability to efficiently control SWs radiation. However, existing schemes still suffer from the bottlenecks of single radiation channel and immutable radiation pattern, which are difficult to satisfy the requirements of high-integration intelligent metadevices. Herein, an ingenious strategy of the SWs radiation metadevice is proposed to independently and dynamically manipulate SWs directional radiation in four polarization channels. The waveguide port and the guided wave structure are designed to excite and propagate the desired SWs, and the programmable coding metasurface can independently convert SWs into *x*-polarized radiation waves, *y*-polarized radiation waves, left-handed circular polarized radiation waves and right-handed circular polarized radiation waves and dynamically control the corresponding radiation angles by adjusting the ON/OFF states of two positive-intrinsic-negative diodes in each spin-decoupled meta-atom. Numerous simulation and experimental results of the proof-of-concept prototype are in good agreement with the theoretical predictions, which verify the feasibility of our proposed methodology. The innovative design of four-channel SWs radiation metadevice with high radiation efficiency and broad radiation bandwidth offers an excellent platform for flexibly manipulating SWs radiation, and possesses tremendous potential in engineering application.

## 1 Introduction

Surface waves (SWs) are special eigen electromagnetic (EM) waves, which are bounded at the interface between two dissimilar media with the EM fields decaying exponentially away from the interface [1], [2], [3], [4]. Multitudinous distinct types of SWs have been discovered and studied in the past few years, such as surface plasmon polaritions (SPP), Bloch SWs, Dyakonov SWs, Tamm waves, etc. [5], [6], [7], [8] Owing to the exotic properties of subwavelength resolution and field localization, SWs have shown a large number of practical applications in antennas, sensors, waveguides, and so on [9], [10], [11], [12]. And in order to better investigate and utilize SWs, some efforts have been devoted to realize efficient conversion between propagating waves (PWs) and SWs [13], [14].

Metasurfaces, artificial planar arrangements of kaleidoscopic subwavelength meta-atoms, possess unprecedented ability to control the phase, amplitude and polarization of EM waves [15], [16], [17]. And given the merits of small size, simple fabrication and exceptional EM performance, metasurfaces gradually replace gratings and prisms to achieve the conversion between SWs and PWs [18], [19], [20]. Especially due to the enormous application potential in wireless communication, the conversion of SWs into customized PWs using metasurfaces has become a research hotspot [21], [22], [23]. For example, on the basis of phase gradient metasurface, an effective method to achieve wideband frequency scanning radiation by transforming SWs was proposed [22]. In addition, by utilizing ultrathin corrugated metallic strips which exhibit the directional radiation property, Xu et al. proposed a kind of spoof surface plasmon polariton (SPP) emitter which can convert SWs into PWs quickly and efficiently [24], [25]. Also, based on the designer Pancharatnam–Berry (PB) metasurface, a series of brand-new strategies which can generate arbitrary spin-polarized scattering far-field patterns from SWs excitations including hologram, vortex beam, and so on were proposed [23], [26]. Furthermore, Wang et al. proposed a spoof SPP leaky-wave antenna made of a periodically modulated SSP waveguide with bilateral 45°-tilted grooves, which can achieve the SWs radiations with arbitrarily customizable polarizations [27]. Nevertheless, existing schemes cannot realize dynamic manipulation of SWs directional radiation in multiple polarization channels, which immensely restrict their practical application in high-integration smart metadevices. Therefore, multichannel SWs radiation metadevices with switchable radiation pattern are imminently needed to further improve the practicability of metasurfaces in SWs radiation.

**Figure 1:**
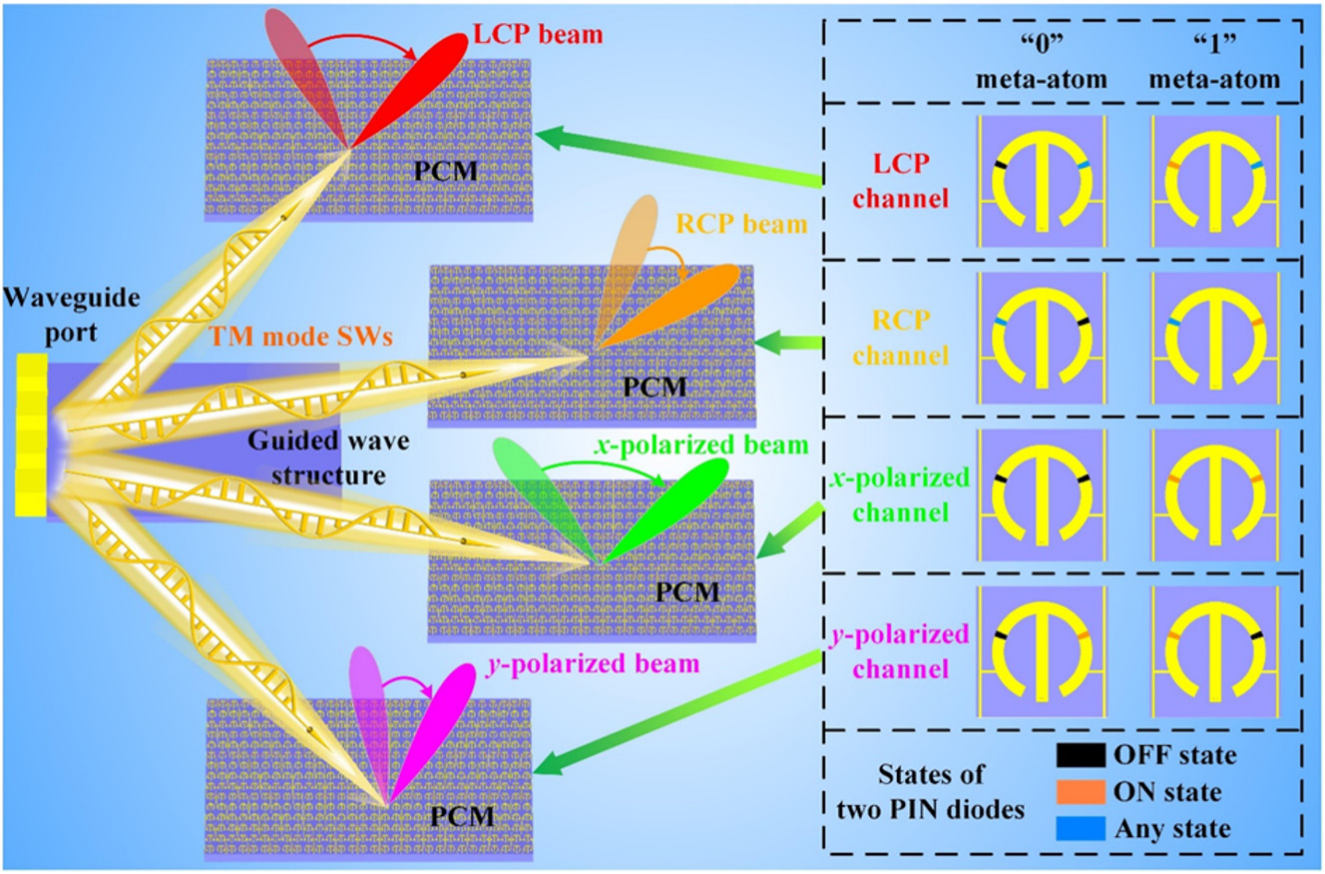
SWs radiation metadevice, which can dynamically control the SWs radiation in four polarization channels. The inset describes the mechanism that the designed spin-decoupled meta-atom achieves 1-bit phase modulation in four polarization channels.

In this work, we propose an appealing paradigm of four-channel SWs radiation metadevice based on the programmable coding metasurface (PCM), which can dynamically control the SWs radiation in four polarization channels, as illustrated in Figure 1. The waveguide port and the guided wave structure are constructed to efficiently excite and propagate the required SWs. By switching the ON/OFF states of two positive-intrinsic-negative (PIN) diodes, the spin-decoupled meta-atom can independently realize the co-polarized reflection for orthogonal circular polarized (CP) waves and *x*-polarized waves and cross-polarized reflection for *x*-polarized waves, and possess 1-bit reflection phases in four channels. Consequently, the PCM composed of the spin-decoupled meta-atoms can convert SWs into left-handed circular polarized (LCP) radiation waves, right-handed circular polarized (RCP) radiation waves, *x*-polarized radiation waves and *y*-polarized radiation waves, and dynamically adjust their radiation angles. Our proposed four-channel SWs radiation metadevice with relatively high radiation efficiency is strongly verified by a series of simulation and experimental results, and exhibits unprecedented flexibility and integration for manipulating SWs directional radiation.

## 2 Design and simulation structural design and mechanism analysis

**Figure 2:**
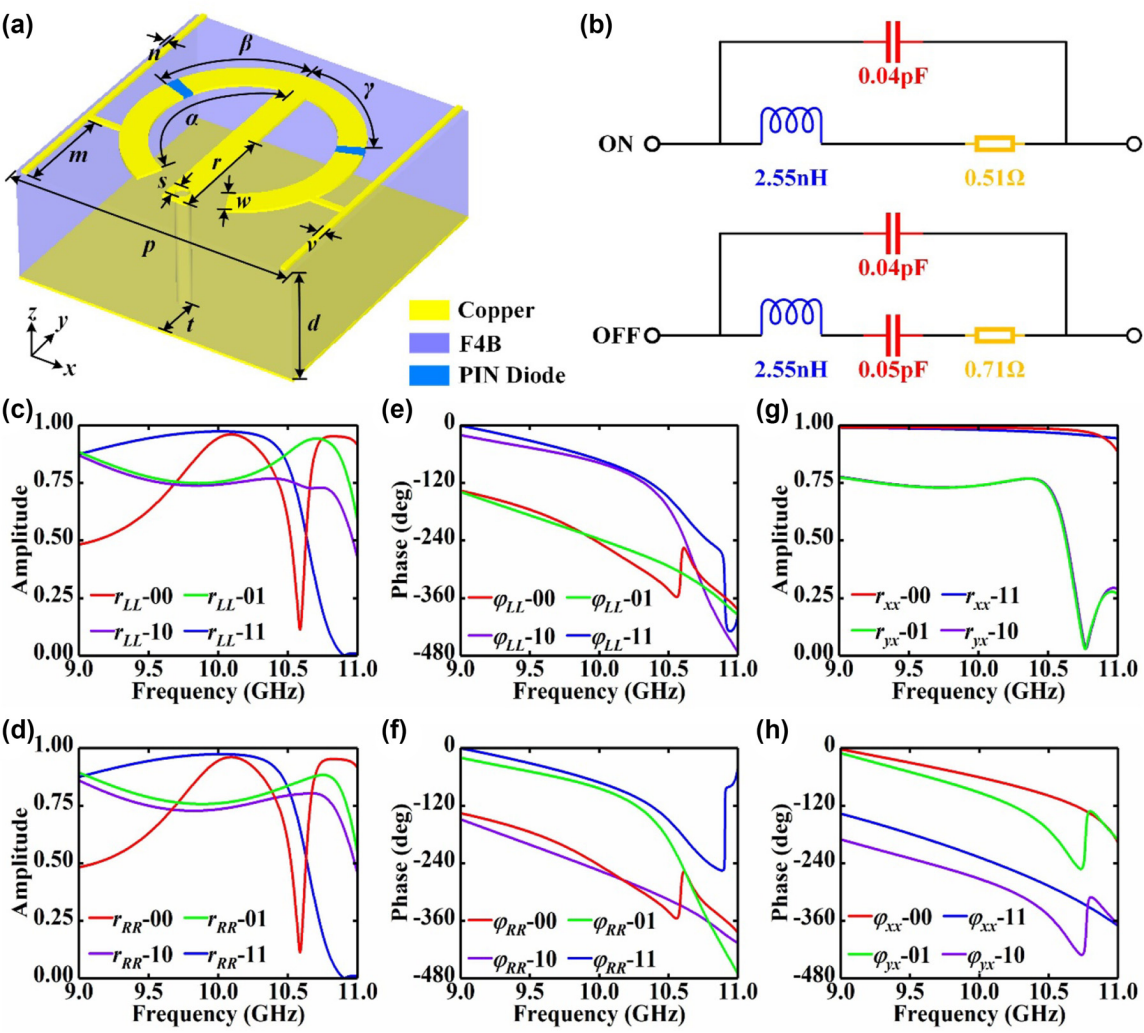
The spin-decoupled meta-atom and the simulated parameters. (a) The schematic diagram of the spin-decoupled meta-atom, where *p* = 10 mm, *d* = 4 mm, *r* = 3.8 mm, *w* = 0.9 mm, *α* = 155°, *β* = 72°, *γ* = 65°, *m* = 3.4 mm, *n* = 0.2 mm, *v* = 0.2 mm, *s* = 0.5 mm, *t* = 1.65 mm. (b) The equivalent circuits of the PIN diode under ON and OFF states. (c)–(d) Simulated co-polarized reflection amplitudes under orthogonal CP incidence. (e)–(f) Simulated co-polarized reflection phases under orthogonal CP incidence. (g) Simulated co-polarized and cross-polarized reflection amplitudes under *x*-polarized incidence. (h) Simulated co-polarized and cross-polarized reflection phases under *x*-polarized incidence.

In order to dynamically control SWs radiation in multiple polarization channels, a spin-decoupled meta-atom composed of the copper structure layer, the F4B dielectric substrate layer (*ε*_*r*_ = 2.65, tan*δ* = 0.001) and the copper sheet layer is meticulously designed, as shown in Figure 2(a). The thickness of the copper structure and the F4B dielectric substrate are 0.017 mm and 4 mm, respectively. The copper structure layer consists of the umbrella-shaped structure and the biasing lines, and the umbrella-shaped structure can be divided into the left arm, the middle handle and the right arm. Two identical PIN diodes (SMP1320 from SKYWORKS) are loaded into the gaps of two arms, and the equivalent circuits of the PIN diode under ON and OFF states are provided in Figure 2(b). A metallized via-hole penetrates from the middle handle to the copper sheet, so the negative sides of two PIN diodes can be connected to the direct current (DC) source through the copper sheet. Furthermore, the positive sides of two PIN diodes are connected with the DC source through the biasing lines. Therefore, the ON and OFF states of two PIN diodes can be independently switched by controlling the external voltage.

With the assistance of the computer simulation technology (CST) microwave studio, the EM responses of the spin-decoupled meta-atom are precisely simulated. For the sake of simplifying the expression, the binary codes “00”, “01”, “10” and “11” are used to represent four different PIN diode states of the meta-atom, where the “1” or “0” in the first bit of the code denotes the “ON” or “OFF” state of the left PIN diode in the meta-atom, and the “1” or “0” in the second bit denotes the “ON” or “OFF” state of the right PIN diode. The simulation results depicted in Figure 2(c) and (d) show that the meta-atom with different PIN diode states can realize efficient co-polarized reflection under orthogonal CP incident waves at 10.3 GHz. What’s more, the co-polarized reflection phase differences between the “10/11” meta-atoms and the “00/01” meta-atoms under LCP incidence are around 180° at 10.3 GHz, and the co-polarized reflection phase differences between the “01/11” meta-atoms and the “00/10” meta-atoms under RCP incidence are also around 180°, as displayed in Figure 2(e) and (f). Thereupon, the spin-decoupled meta-atom is able to generate 1-bit co-polarized reflection phases for LCP waves and RCP waves by switching the ON/OFF states of the left PIN diode and the right PIN diode, respectively.

The EM responses of the spin-decoupled meta-atom under linear polarized (LP) incident waves can be analyzed using the Jones matrix [28], [29]. Due to the cross-polarized reflection amplitudes of the meta-atom under orthogonal CP incident waves approach 0 at 10.3 GHz, the relationship between CP reflected waves and CP incident waves can be written as follow:

(1) $$\left(\begin{array}{c} E_{L}^{r}\\ E_{R}^{r} \end{array}\right)=R_{CP}\cdot \left(\begin{array}{c} E_{L}^{i}\\ E_{R}^{i} \end{array}\right)=\left(\begin{array}{cc} R_{LL} & 0\\ 0 & R_{RR} \end{array}\right)\cdot \left(\begin{array}{c} E_{L}^{i}\\ E_{R}^{i} \end{array}\right)$$

Here, $E_{L}^{r}$ ( $E_{R}^{r}$ ) and $E_{L}^{i}$ ( $E_{R}^{i}$ ) denote LCP (RCP) reflected waves propagating along +*z* direction and LCP (RCP) incident waves propagating along −*z* direction, respectively. $R_{LL}=r_{LL}\cdot {\text{e}}^{\text{i}\cdot \varphi_{LL}}$ ( $R_{RR}=r_{RR}\cdot {\text{e}}^{\text{i}\cdot \varphi_{RR}}$ ) represents the co-polarized reflection coefficient under LCP (RCP) incidence, where *r*_*LL*_ (*r*_*RR*_) and *φ*_*LL*_ (*φ*_*RR*_) are the co-polarized reflection amplitude and phase under LCP (RCP) incidence. Moreover, the relationship between LP reflected waves and LP incident waves can be expressed as follow:

(2) $$\left(\begin{array}{c} E_{x}^{r}\\ E_{y}^{r} \end{array}\right)=R_{LP}\cdot \left(\begin{array}{c} E_{x}^{i}\\ E_{y}^{i} \end{array}\right)=\left(\begin{array}{cc} R_{xx} & R_{xy}\\ R_{yx} & R_{yy} \end{array}\right)\cdot \left(\begin{array}{c} E_{x}^{i}\\ E_{y}^{i} \end{array}\right)$$

Here, $E_{x}^{r}$ ( $E_{y}^{r}$ ) and $E_{x}^{i}$ ( $E_{y}^{i}$ ) denote *x*-polarized (*y*-polarized) reflected waves propagating along +*z* direction and *x*-polarized (*y*-polarized) incident waves propagating along −*z* direction, respectively. $R_{xx}=r_{xx}\cdot {\text{e}}^{\text{i}\cdot \varphi_{xx}}$ ( $R_{yy}=r_{yy}\cdot {\text{e}}^{\text{i}\cdot \varphi_{yy}}$ ) and $R_{yx}=r_{yx}\cdot {\text{e}}^{\text{i}\cdot \varphi_{yx}}$ ( $R_{xy}=r_{xy}\cdot {\text{e}}^{\text{i}\cdot \varphi_{xy}}$ ) represent the co-polarized and cross-polarized reflection coefficients under *x*-polarized (*y*-polarized) incidence. According to the relationship between CP waves and LP waves, CP reflected waves and CP incident waves can also be described as follow:

(3) $$\left(\begin{array}{c} E_{L}^{r}\\ E_{R}^{r} \end{array}\right)=\left(\begin{array}{cc} 1 & i\\ 1 & -i \end{array}\right)\cdot \left(\begin{array}{c} E_{x}^{r}\\ E_{y}^{r} \end{array}\right)/\sqrt{2}$$

(4) $$\left(\begin{array}{c} E_{L}^{i}\\ E_{R}^{i} \end{array}\right)=\left(\begin{array}{cc} 1 & -i\\ 1 & i \end{array}\right)\cdot \left(\begin{array}{c} E_{x}^{i}\\ E_{y}^{i} \end{array}\right)/\sqrt{2}$$

Based on Equation (1)–(4), the relationship between *R*_*LP*_ and *R*_*CP*_ is derived as follow:

(5) $$R_{LP}={\left(\begin{array}{cc} 1 & i\\ 1 & -i \end{array}\right)}^{-1}\cdot R_{CP}\cdot \left(\begin{array}{cc} 1 & -i\\ 1 & i \end{array}\right)$$

At 10.3 GHz, *r*_*LL*_ and *r*_*RR*_ can be regarded as *r*_*LL*_ ≈ *r*_*RR*_ ≈ 1 in the light of the simulation results, and the difference between *φ*_*RR*_ and *φ*_*LL*_ can be defined as Δ*φ* = *φ*_*RR*_ − *φ*_*LL*_, so Equation (5) will be transformed as follow:

(6) $$\left(\begin{array}{cc} R_{xx} & R_{xy}\\ R_{yx} & R_{yy} \end{array}\right)=\left(\begin{array}{cc} \left(1+{\text{e}}^{\text{i}\cdot \Delta \varphi }\right){\text{e}}^{\text{i}\cdot \varphi_{LL}} & \left(1-{\text{e}}^{\text{i}\cdot \Delta \varphi }\right){\text{e}}^{\text{i}\cdot \left(\varphi_{LL}-\pi /2\right)}\\ \left(1-{\text{e}}^{\text{i}\cdot \Delta \varphi }\right){\text{e}}^{\text{i}\cdot \left(\varphi_{LL}-\pi /2\right)} & \left(1+{\text{e}}^{\text{i}\cdot \Delta \varphi }\right){\text{e}}^{\text{i}\cdot \left(\varphi_{LL}+\pi \right)} \end{array}\right)/2$$

It can be calculated from Equation (6) that $\left(\begin{array}{cc} R_{xx} & R_{xy}\\ R_{yx} & R_{yy} \end{array}\right)=\left(\begin{array}{cc} {\text{e}}^{\text{i}\cdot \varphi_{LL}} & 0\\ 0 & {\text{e}}^{\text{i}\cdot \left(\varphi_{LL}+\pi \right)} \end{array}\right)$ when Δ*φ* = 0 and $\left(\begin{array}{cc} R_{xx} & R_{xy}\\ R_{yx} & R_{yy} \end{array}\right)=\left(\begin{array}{cc} 0 & {\text{e}}^{\text{i}\cdot \left(\varphi_{LL}-\pi /2\right)}\\ {\text{e}}^{\text{i}\cdot \left(\varphi_{LL}-\pi /2\right)} & 0 \end{array}\right)$ when Δ*φ* = *π*, which suggest that the spin-decoupled meta-atom will reflect *x*-polarized waves into co-polarized waves when the ON/OFF states of two PIN diodes are identical and reflect *x*-polarized waves into cross-polarized waves when the ON/OFF states of two PIN diodes are different. Besides, the meta-atom is able to achieve 1-bit co-polarized phase modulation for *x*-polarized waves by simultaneously adjusting two PIN diodes with identical ON/OFF states and achieve 1-bit cross-polarized phase modulation by simultaneously adjusting two PIN diodes with different ON/OFF states. The simulation results plotted in Figure 2(g) and (h) indicate that the spin-decoupled meta-atom can realize efficient co-polarized and cross-polarized reflection under *x*-polarized incidence and generate 1-bit reflection phases, which strongly demonstrate the aforementioned theoretical analysis.

**Figure 3:**
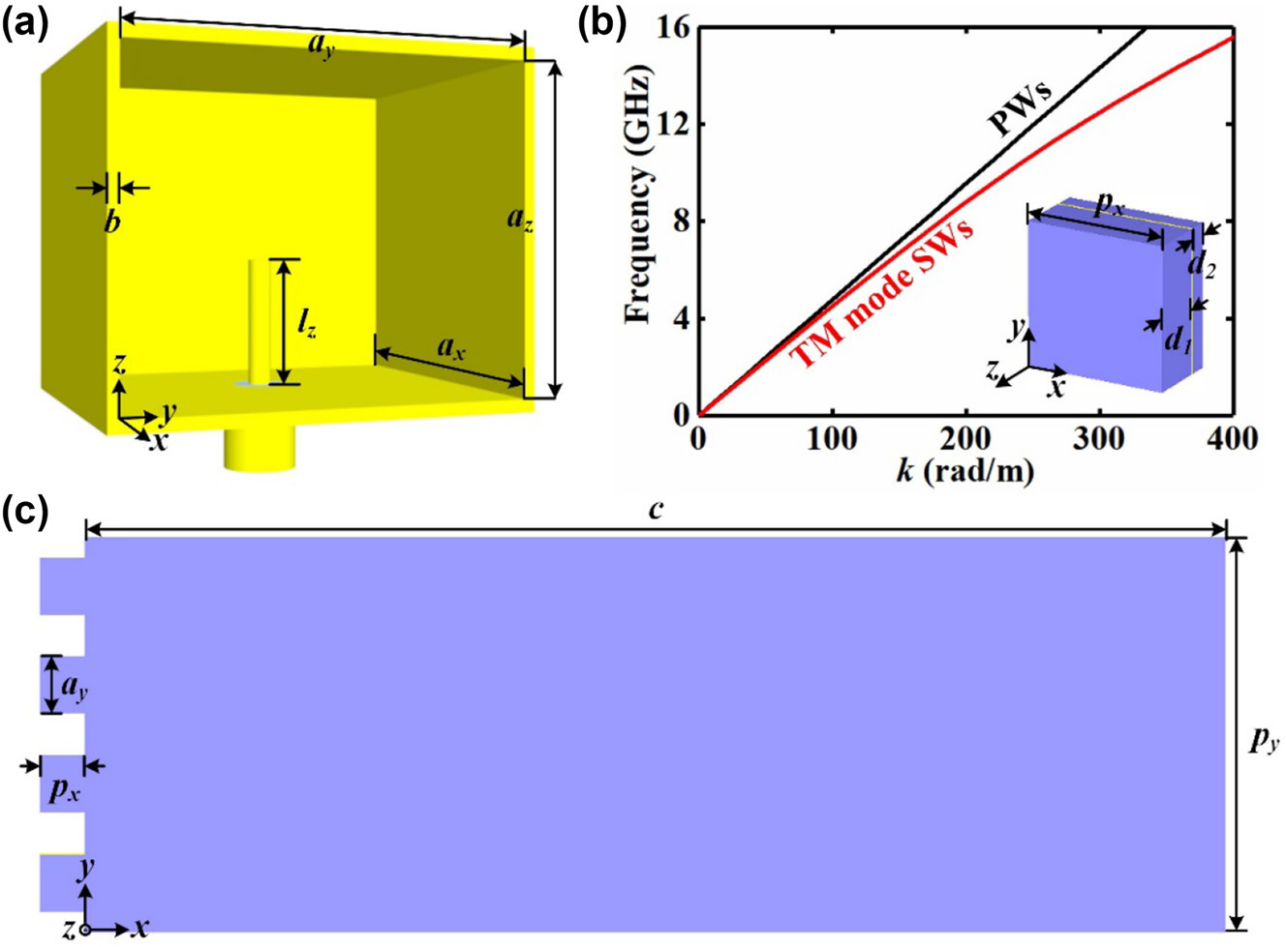
The guided wave structure. (a) The sketch map of the waveguide port, where *a*_*x*_ = 19 mm, *a*_*y*_ = 21.8 mm, *a*_*z*_ = 14.8 mm, *b* = 1.5 mm, *l*_*z*_ = 6 mm. (b) Simulated dispersion relation of the guided wave meta-atom. The inset describes the guided wave meta-atom, where *p*_*x*_ = 7.5 mm, *d*_1_ = 3 mm, *d*_2_ = 1 mm. (b) The schematic diagram of the guided wave structure, where *c* = 187.5 mm, *p*_*y*_ = 150 mm.

As shown in Figure 3(a), the waveguide port excited by the monopole antenna is judiciously constructed to launch EM waves. And the guided wave structure composed of the guided wave meta-atoms is designed to convert the EM waves emitted by the waveguide port into TM mode SWs and stably propagate TM mode SWs to the PCM. The guided wave meta-atom illustrated in Figure 3(b) consists of one copper sheet layer and two F4B dielectric substrate layers. The thickness of the copper sheet layer is 0.017 mm, and the thickness of two F4B dielectric substrate layers is 3 mm and 1 mm, respectively. The simulated dispersion relation of the guided wave meta-atom suggests that the wave vector of the supported TM mode SWs *k*_SWs_ is larger than that of the PWs in free space *k*_0_, and *k*_SWs_ = 1.09*k*_0_ at 10.3 GHz, as depicted in Figure 3(b). Based on the guided wave meta-atom, the guided wave structure is constructed to connect to four waveguide ports and the PCM, as portrayed in Figure 3(c).

## 3 Design and simulation of four-channel SWs radiation metadevice

According to the previous theoretical analysis and structural design, a SWs radiation metadevice composed of the PCM, the guided wave structure and four waveguide ports is established to dynamically manipulate the SWs radiation pattern in multiple polarization channels. The PCM consists of 30 × 15 identical spin-decoupled meta-atoms. Because the proposed spin-decoupled meta-atom can achieve efficient co-polarized and cross-polarized reflection for *x*-polarized waves, the PCM is capable of converting TM mode SWs into *x*-polarized and *y*-polarized radiation waves. In addition, TM mode SWs can be regarded as the superposition of LCP SWs and RCP SWs, so the PCM can also independently manipulate the radiation of orthogonal CP SWs. Consequently, the established SWs radiation metadevice is able to dynamically controlling the SWs radiation pattern in *x*-polarized channel, *y*-polarized channel, LCP channel and RCP channel. To simplify the expression, the code “0” and code “1” are used to denote the phases of 0° and 180°, respectively. The phase of the PCM in the *y* direction is constant, and the PCM should provide the phase gradient ∇*φ* opposite to the wave vector *k*_SWs_ in the *x* direction to reduce the *k*_SWs_ and achieve SWs directional radiation. On the basis of the generalized Snell’s law [30], [31], the radiation angle *θ*_*r*_ of SWs is calculated as follow:

(7) $$k_{0}\sin\left(\theta_{r}\right)=k_{\text{SWs}}\sin\left(\theta_{i}\right)+\nabla \varphi$$

**Figure 4:**
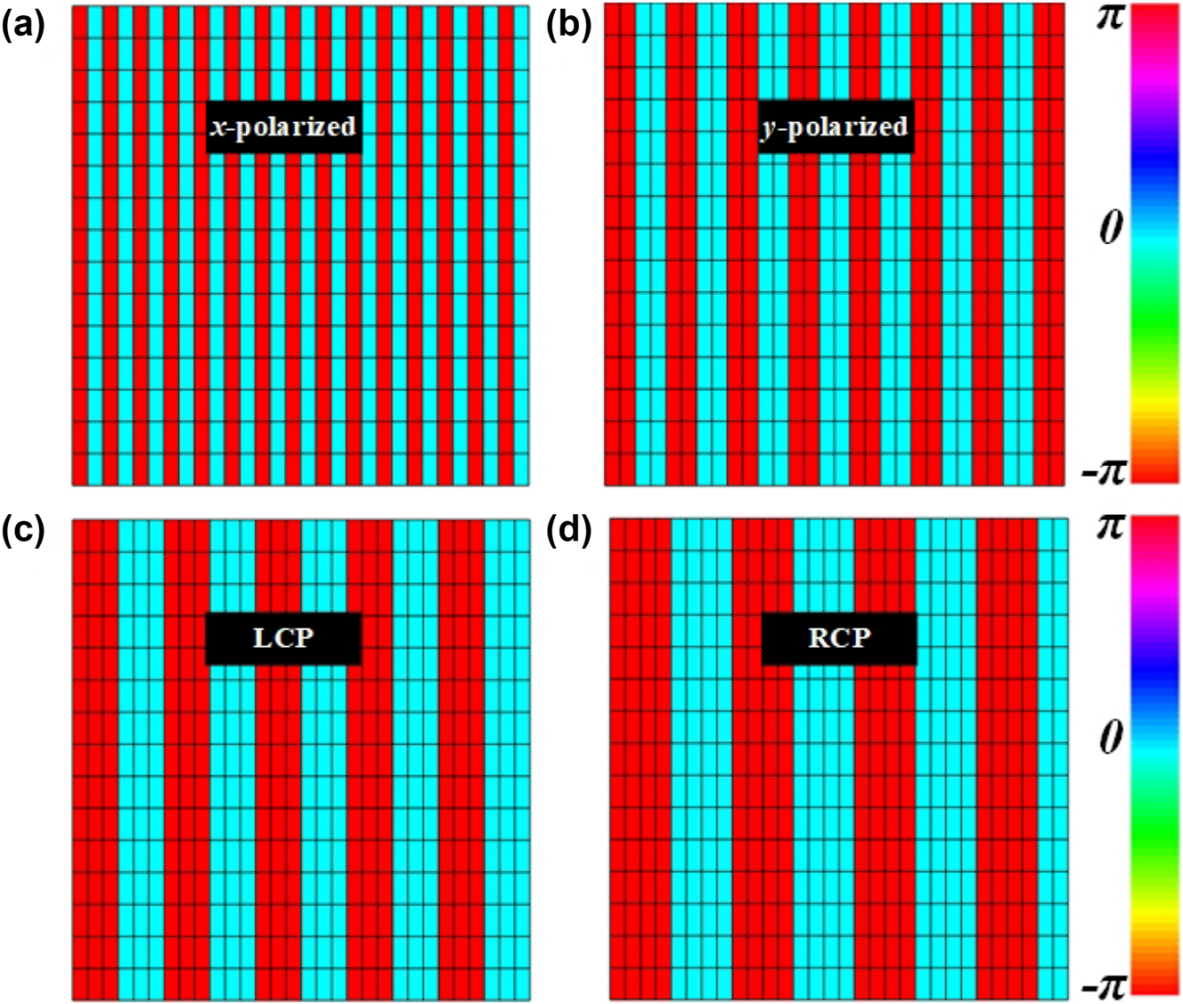
The reflection phases of part of spin-decoupled meta-atoms of the SWs radiation metadevice in (a) *x*-polarized channel, (b) *y*-polarized channel, (c) LCP channel, (d) RCP channel.

Here, the incident angle *θ*_*i*_ of SWs is *θ*_*i*_ = 90°, and the phase gradient ∇*φ* can be written as ∇*φ* = d*φ*/d*x*, where d*x* is the period of a group of the spin-decoupled meta-atoms with the same phase, and the phase difference d*φ* between two adjacent groups of meta-atoms is d*φ* = *π* in the proposed PCM. In our design, the PCM consists of 30 × 15 phase modulation meta-atoms, and the meta-atoms in the *y* direction are completely the same because the phase of the metasurface in the *y* direction is constant. Two groups of verification schemes are provided to demonstrate our methodology. In the first verification scheme, the phase coding sequences of the PCM in *x*-polarized channel, *y*-polarized channel, LCP channel and RCP channel are “01010101…”, “00110011…”, “000111000…” and “00001111…”, and the corresponding reflection phase distributions of the PCM at 10.3 GHz are described in Figure 4(a)–(d). Based on Equation (7), the radiation angles of *x*-polarized radiation waves, *y*-polarized radiation waves, LCP radiation waves and RCP radiation waves are calculated as −21.5°, 21.2°, 37.2° and 46.5° at 10.3 GHz.

**Figure 5:**
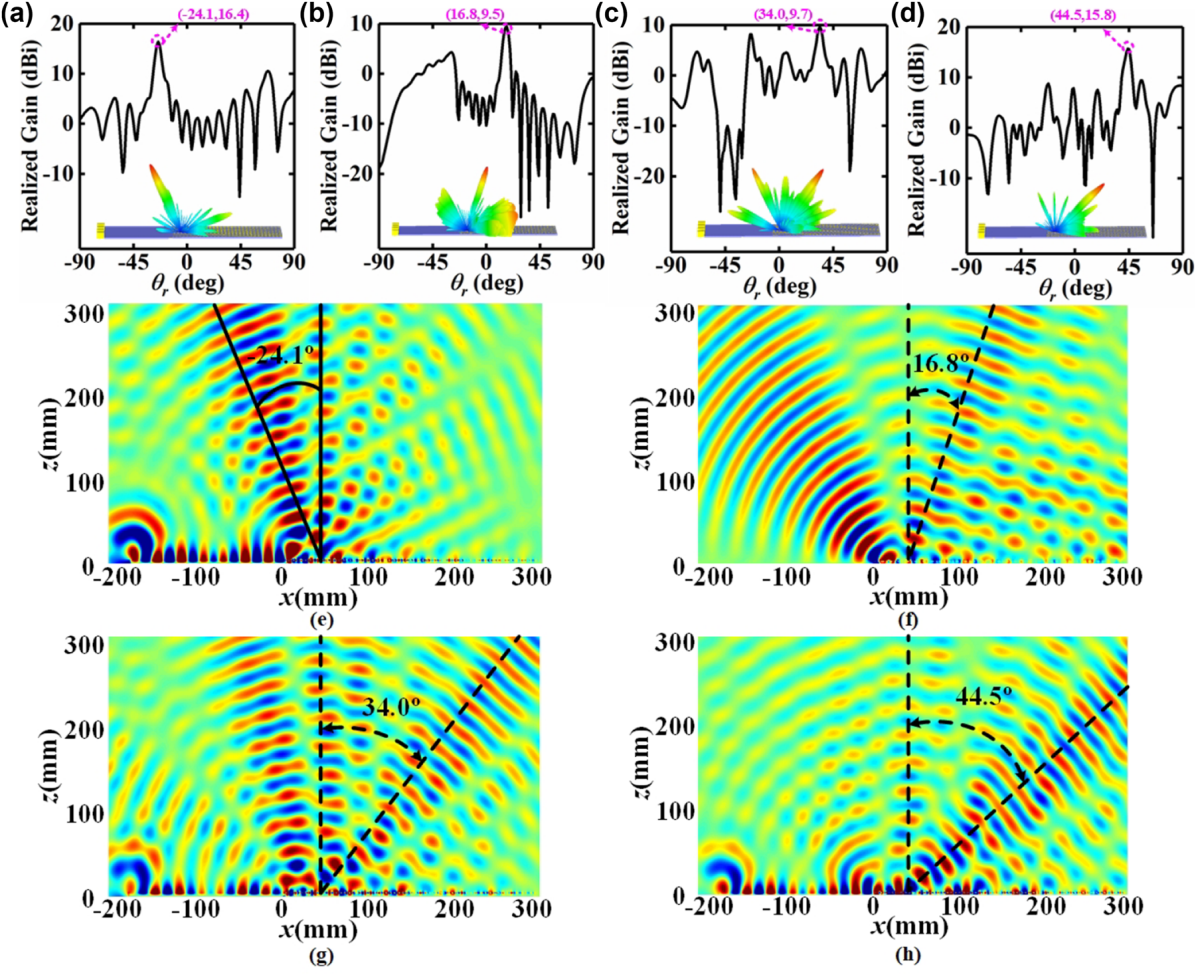
Simulated realized gain and 3D radiation pattern of the SWs radiation metadevice in (a) *x*-polarized channel, (b) *y*-polarized channel, (c) LCP channel, (d) RCP channel. Simulated electric field distribution of the SWs radiation metadevice in (e) *x*-polarized channel, (f) *y*-polarized channel, (g) LCP channel, (h) RCP channel.

As displayed in Figure 5(a), the far-field simulation result in *x*-polarized channel indicates that a high-gain beam with the radiation angle of −24.1° is radiated by the SWs radiation metadevice at 10.3 GHz, and the radiation efficiency and total efficiency are 92.8 % and 82.1 % respectively. Besides, we can intuitively observe from the near-field simulation result in Figure 5(e) that the *x*-polarized SWs flow across the guided wave structure and are converted into the *x*-polarized radiation waves by the PCM at a radiation angle of −24.1°, which is consistent with previous theoretical prediction. As exhibited in Figure 5(b) and (f), a beam of *y*-polarized radiation waves with the radiation angle of 16.8° is generated by the SWs radiation metadevice, and the radiation efficiency and total efficiency are 93.3 % and 83.2 % respectively. Furthermore, the LCP SWs are converted into LCP radiation waves with the radiation angle of 53.0°, and the RCP SWs are converted into RCP radiation waves with the radiation angle of 57.9°, as shown in Figure 5(c) and (d) and (g) and (h). The radiation efficiencies in LCP channel and RCP channel are 88.6 % and 81.1 %, and the total efficiencies in two channels are 82.6 % and 69.7 %.

**Figure 6:**
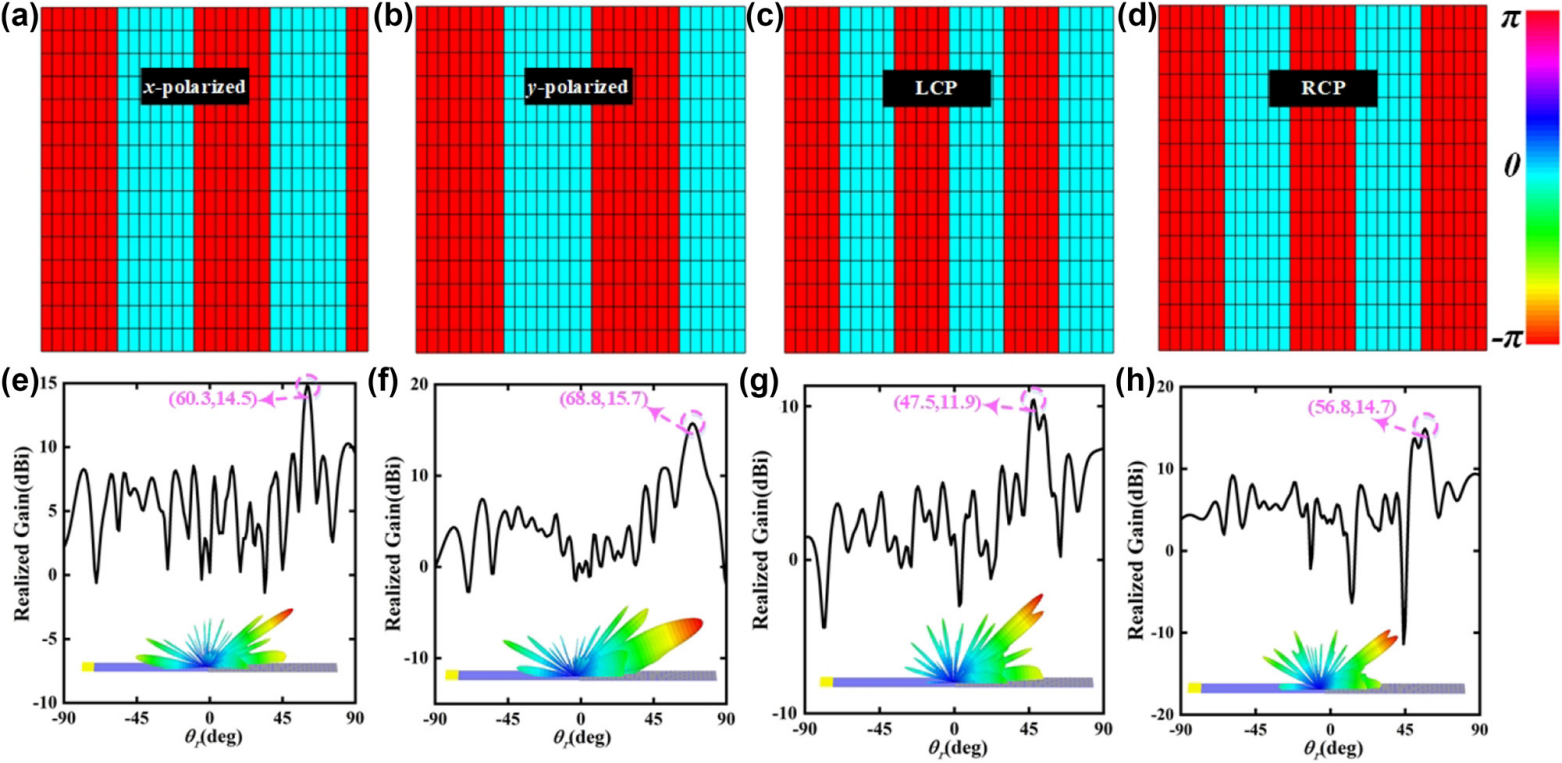
The reflection phases of part of spin-decoupled meta-atoms of the SWs radiation metadevice in (a) *x*-polarized channel, (b) *y*-polarized channel, (c) LCP channel, (d) RCP channel. Simulated realized gain and 3D radiation pattern of the SWs radiation metadevice in (e) *x*-polarized channel, (f) *y*-polarized channel, (g) LCP channel, (h) RCP channel.

Furthermore, for another verification scheme, the phase coding sequences of the PCM in *x*-polarized channel, *y*-polarized channel, LCP channel and RCP channel are “00000001111111…”, “0000000011111111…”, “0000011111…” and “000000111111…”, and the corresponding reflection phase distributions of the PCM at 10.3 GHz are described in Figure 6(a)–(d). On the basis of Equation (7), the radiation angles of *x*-polarized radiation waves, *y*-polarized radiation waves, LCP radiation waves and RCP radiation waves are calculated as 61.9°, 68.1°, 53.0° and 57.9° at 10.3 GHz.

As displayed in Figure 6(e), the far-field simulation result in *x*-polarized channel indicates that a high-gain beam with the radiation angle of 60.3° is radiated by the SWs radiation metadevice at 10.3 GHz, and the radiation efficiency and total efficiency are 85.4 % and 79.7 % respectively. As exhibited in Figure 6(f), a beam of *y*-polarized radiation waves with the radiation angle of 68.8° is generated by the SWs radiation metadevice, and the radiation efficiency and total efficiency are 93.6 % and 81.6 % respectively. Furthermore, the LCP SWs are converted into LCP radiation waves with the radiation angle of 47.5°, and the RCP SWs are converted into RCP radiation waves with the radiation angle of 56.8°, as shown in Figure 6(g) and (h). The radiation efficiencies in LCP channel and RCP channel are 82.8 % and 85.7 %, and the total efficiencies in two channels are 82.8 % and 79.2 %. All in all, the simulation results demonstrate that the designed SWs radiation metadevice can independently and dynamically control the SWs radiation with high efficiency in four polarization channels.

**Figure 7:**
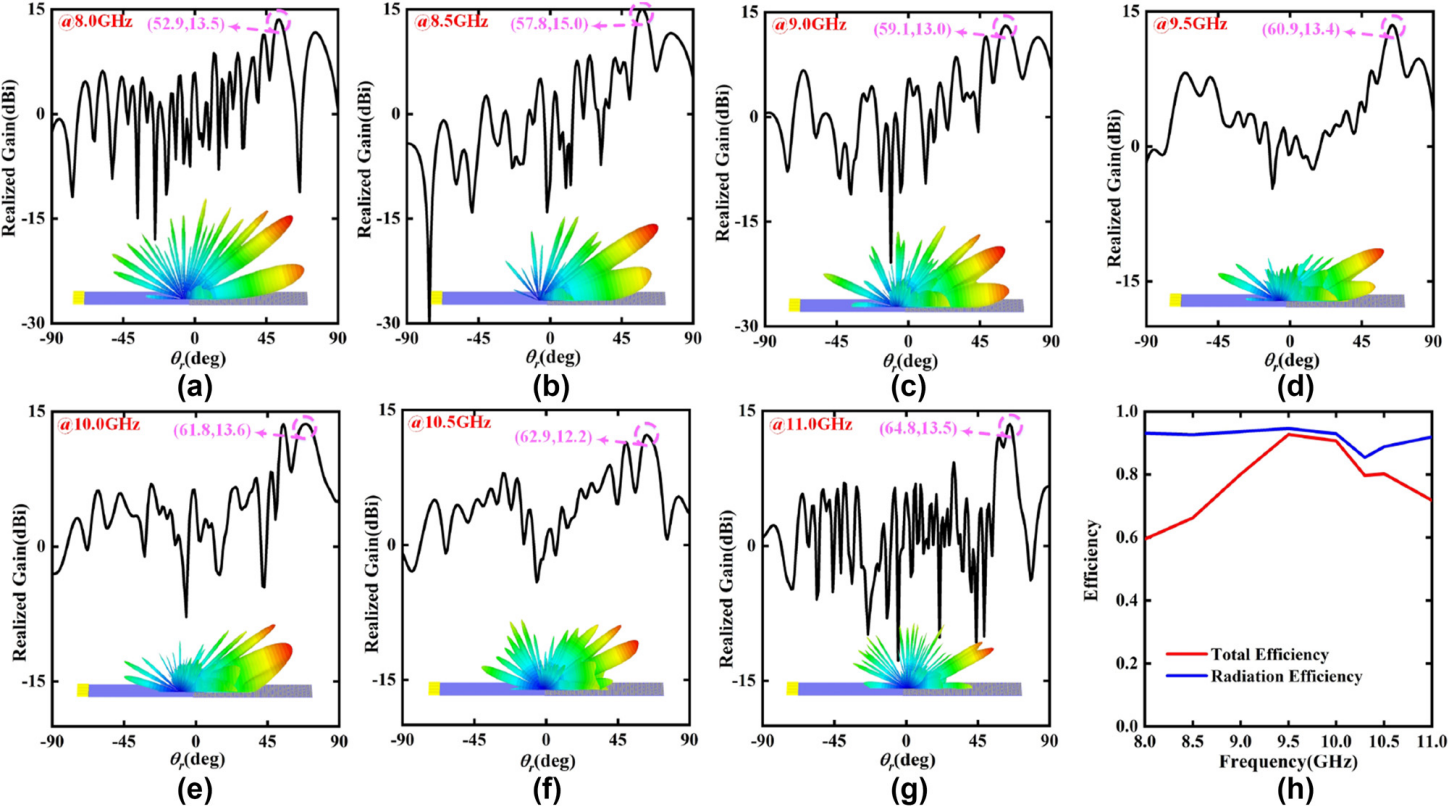
The simulated realized gain and 3D radiation pattern of the SWs radiation metadevice with the phase coding sequence “00000001111111…” for *x*-polarized channel at different frequency points: (a) 8.0 GHz, (b) 8.5 GHz, (c) 9.0 GHz, (d) 9.5 GHz, (e) 10.0 GHz, (f) 10.5 GHz, (g) 11.0 GHz. (h) The radiation and total efficiencies of the SWs radiation metadevice in the frequency band from 8.0 GHz to 11.0 GHz.

Also, the broadband performance of the SWs radiation metadevice is evaluated. As an example, the far-field simulation results of the PCM with the phase coding sequence “00000001111111…” for *x*-polarized channel, are displayed in Figure 7(a)–(g). The simulation results show that a high-gain beam with different radiation angles is radiated by the SWs radiation metadevice in a wide bandwidth from 8.0 GHz to 11.0 GHz, which are in excellent accordance with our theoretical predictions in Equation (7). In addition, the radiation and the total efficiencies of the SWs radiation metadevice in the 8.0 GHz–11.0 GHz band exceed 85.0 % and 60.0 %, respectively, as shown in Figure 7(h). The above simulation results show that the designed SWs radiation metadevice can independently and dynamically control the SWs radiation with high efficiency over a wide bandwidth from 8.0 GHz to 11.0 GHz.

## 4 Experimental verification

**Figure 8:**
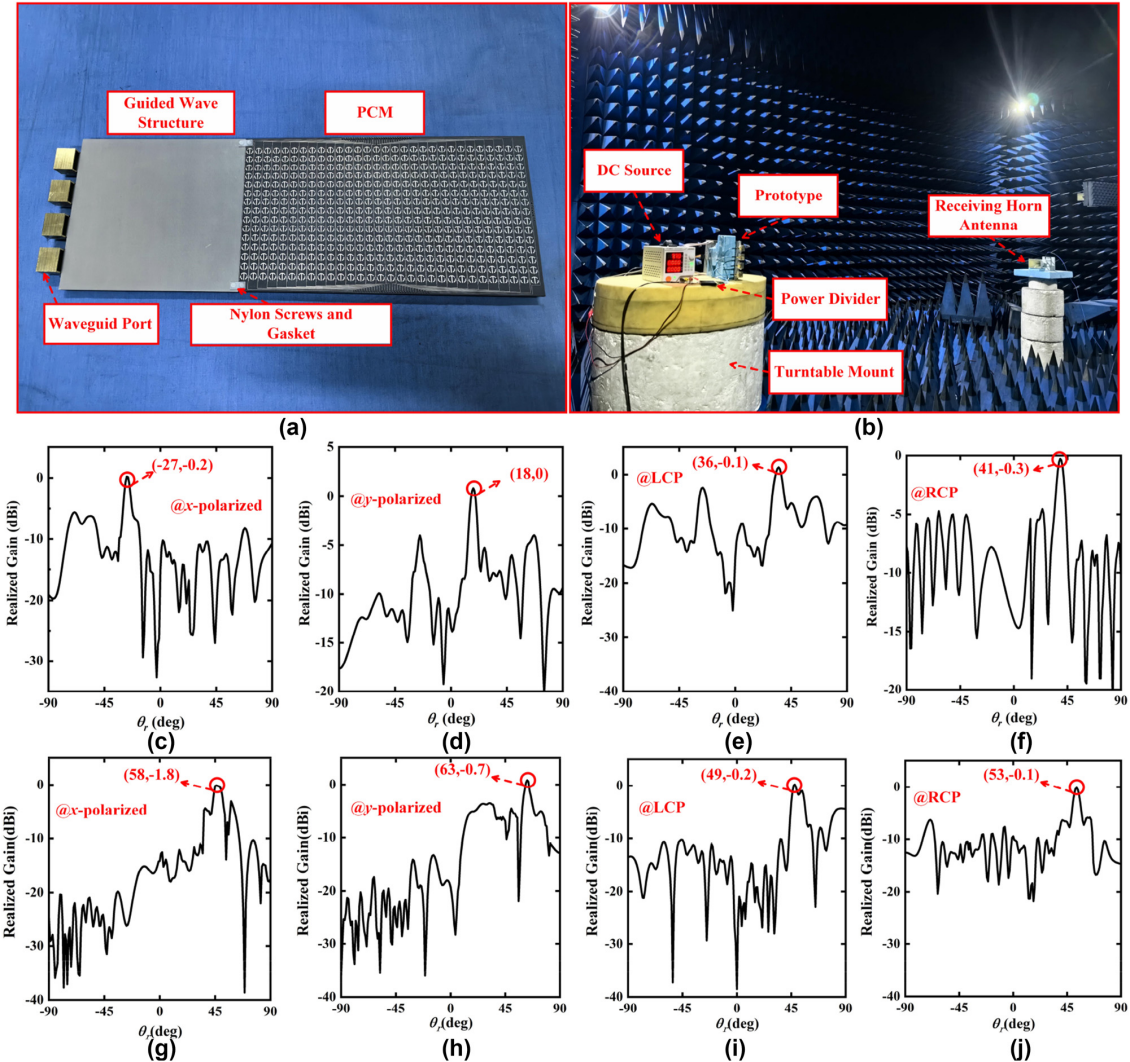
The far-field experiments and the corresponding results. (a) Fabricated prototypes of proposed SWs radiation metadevices. (b) The far-field experimental setups. The normalized experimental results of the PCM with the first-group phase coding sequences “01010101…”, “00110011…”, “000111000…” and “00001111…” for (c) *x*-polarized channel, (d) *y*-polarized channel, (e) LCP channel and (f) RCP channel. The normalized experimental results of the PCM with another group phase coding sequences “00000001111111…”, “0000000011111111…”, “0000011111…” and “000000111111…” for (g) *x*-polarized channel, (h) *y*-polarized channel, (i) LCP channel and (j) RCP channel.

As exhibited in Figure 8(a), in order to verify the practicability of our proposed schemes, by utilizing the low-cost printed circuit board (PCB) technology, the elaborate prototype of the SWs radiation metadevice identical to the simulation model is fabricated. The fabricated prototypeconsists of 30 × 15 phase modulation meta-atoms. By inserting four waveguide ports into the guided wave structure, and connecting the PCM with the guided wave structure, the SWs radiation metadevice is assembled with modular design and low production cost. In the microwave anechoic chamber, the far-field experiments are implemented and the experimental setups are demonstrated in Figure 8(b). Through a power divider, the four waveguide ports are connected to port 1 of a vector network analyzer (VNA), and the receiving horn antenna is placed far enough from the SWs radiation metadevice and connected to port 2 of the VNA. The radiation field intensity distribution of the SWs radiation metadevice at different angles can be measured by rotating the metadevice on the turntable mount. Figure 8(c)–(f) show the normalized experimental results of the PCM with the first-group phase coding sequences “01010101…”, “00110011…”, “000111000…” and “00001111…” for *x*-polarized channel, *y*-polarized channel, LCP channel and RCP channel. The experimental results show that a high-gain beam with different radiation angles is radiated by the SWs radiation metadevice, which are in good accordance with the simulation results in Figure 5. Likewise, the normalized experimental results of the PCM with another group phase coding sequences “00000001111111…”, “0000000011111111…”, “0000011111…” and “000000111111…” for *x*-polarized channel, *y*-polarized channel, LCP channel and RCP channel are illustrated in Figure 8(g)–(j), which are in good accordance with the simulation results in Figure 6 as well. The two phase coding sequences of the SWs radiation metadevice are dynamically switched by altering the state of two PIN diodes of each phase dynamic modulation meta-atom, and the experimental results described in Figure 8(c)–(j) suggest that the radiation angle of SWs can be flexibly adjusted in four channels according to the preset design at 10.3 GHz.

**Figure 9:**
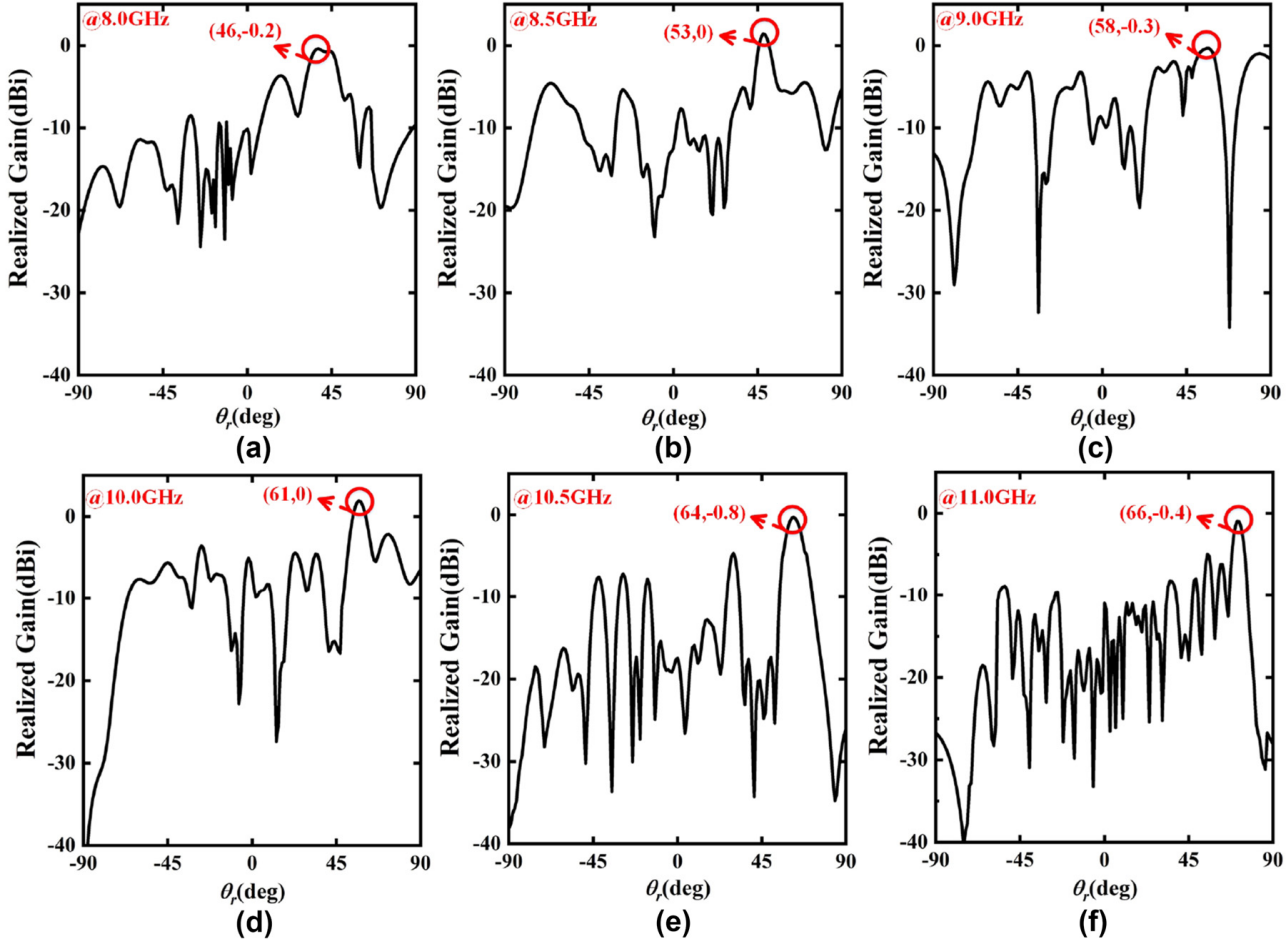
The normalized experimental results of the PCM with the phase coding sequence “00000001111111…” for *x*-polarized channel at different frequency points: (a) 8.0 GHz, (b) 8.5 GHz, (c) 9.0 GHz, (d) 10.0 GHz, (e) 10.5 GHz, (f) 11.0 GHz.

Furthermore, the broadband performance of the SWs radiation metadevice is measured. The normalized experimental results of the PCM with the phase coding sequence “00000001111111…” for *x*-polarized channel, are displayed in Figure 9(a)–(f). The experimental results show that the SWs radiation metadevice can realize beam scanning from 46° to 66° in the *x*-polarized channel, which are consistent with the theoretical results and simulation results. For the same reason, the remaining three channels have the same broadband beam scanning performance. Undeniably, there are some deviations between the experimental results and the simulation results, mainly due to the inaccurate manual operations of the experimental setups and the unavoidable sample fabrication errors in PCB processing and welding. Overall, the experimental results are in general agreement with the theoretical and simulation results, which further strongly demonstrates the effectiveness of our proposed methodology.

## 5 Conclusions

In this paper, we propose an inspiring design of the SWs radiation metadevice to independently realize dynamic manipulation of SWs directional radiation in four polarization channels. The waveguide port and the guided wave structure are able to excite and propagate TM mode SWs. By adjusting the ON/OFF states of the PIN diodes, the PCM can independently achieve SWs directional radiation in *x*-polarized channel, *y*-polarized channel, LCP channel and RCP channel, and dynamically control the radiation angle of the radiation beam in each channel. Multitudinous simulations and experiments corroborate the feasibility of our proposed methodology, and show that the designed SWs radiation metadevice possesses efficient and broadband performance in four polarization channels. We firmly believe that our proposed paradigm opens a brand-new avenue for constructing SWs radiation metadevice with higher flexibility and serviceability, and has tremendous application potential in sensing, communication and other multifunctional smart metadevices.
